# *De Novo* Brain-Computer Interfacing Deforms Manifold of Populational Neural Activity Patterns in Human Cerebral Cortex

**DOI:** 10.1523/ENEURO.0145-22.2022

**Published:** 2022-11-23

**Authors:** Seitaro Iwama, Yichi Zhang, Junichi Ushiba

**Affiliations:** 1 School of Fundamental Science and Technology, Graduate School of Keio University, Kanagawa, 223-8522, Japan; 2 Japan Society for the Promotion of Science, Tokyo, 102-0083, Japan; 3 Department of Biosciences and Informatics, Faculty of Science and Technology, Keio University, Kanagawa, 223-8522, Japan

**Keywords:** brain-computer interface, *de novo* learning, neural plasticity, nonlinear dimensionality reduction, sensorimotor activity

## Abstract

Human brains are capable of modulating innate activities to adapt to novel environments and tasks; for sensorimotor neural system this means acquisition of a rich repertoire of activity patterns that improve behavioral performance. To directly map the process of acquiring the neural repertoire during tasks onto performance improvement, we analyzed net neural populational activity during the learning of its voluntary modulation by brain-computer interface (BCI) operation in female and male humans. The recorded whole-head high-density scalp electroencephalograms (EEGs) were subjected to dimensionality reduction algorithm to capture changes in cortical activity patterns represented by the synchronization of neuronal oscillations during adaptation. Although the preserved variance of targeted features in the reduced dimensions was 20%, we found systematic interactions between the activity patterns and BCI classifiers that detected motor attempt; the neural manifold derived in the embedded space was stretched along with motor-related features of EEG by model-based fixed classifiers but not with adaptive classifiers that were constantly recalibrated to user activity. Moreover, the manifold was deformed to be orthogonal to the boundary by *de novo* classifiers with a fixed decision boundary based on biologically unnatural features. Collectively, the flexibility of human cortical signaling patterns (i.e., neural plasticity) is only induced by operation of a BCI whose classifier required fixed activities, and the adaptation could be induced even the requirement is not consistent with biologically natural responses. These principles of neural adaptation at a macroscopic level may underlie the ability of humans to learn wide-ranging behavioral repertoires and adapt to novel environments.

## Significance Statement

We investigated adaption of macroscopic neural activities during brain-computer interface (BCI) operation to directly map the process of acquiring the neural repertoire for performance improvement. When the classifier incorporated in BCI was fixed and based on the desynchronization of neural oscillations, the distribution of activity patterns (neural manifold) showed the improved separability along with the motor-related component of electroencephalograms (EEGs) to improve BCI controllability. Meanwhile the adaptive classifier constantly fitted to current user activity did not elicit such adaptation of neural activity patterns. Moreover, even the classifiers based on biologically unnatural model induced the adaptation, captured by deformation of neural manifold. Neural adaptation processes at a macroscopic level may underlie the ability of humans to learn wide-ranging behavioral repertoires.

## Introduction

Human beings can sophisticate motor plans and subsequent actions to dynamically interact with the external environment (Burdet et al., 2001; Todorov and Jordan, 2002; Scott, 2004). One surprising demonstration is an adaptation to changes in the properties of physical interfaces such as the use of novel tools or loss and augmentation of body parts by tuning distributed sensorimotor circuitries to achieve smooth interaction with surroundings (Imamizu et al., 2000; Quallo et al., 2009; Penaloza and Nishio, 2018; Mehring et al., 2019; Choi et al., 2020; Kieliba et al., 2021; Rossi et al., 2021).

The internal representation of sensorimotor adaptation has been sought by electrophysiology and neuroimaging techniques (Karni et al., 1995; Nudo et al., 1996; Kleim et al., 2004; Diedrichsen et al., 2005; Berlot et al., 2020). In particular, the primary motor cortex (M1) exhibits covariance patterns of multiple neural units, namely, the neural manifold which reliably represents ongoing behavior and its correction (Gallego et al., 2018, 2020; Perich et al., 2018; Shenoy and Kao, 2021). Moreover, direct mapping of behavior and single neuron activity patterns achieved with brain-computer interfaces (BCIs) revealed monkeys are capable of endogenous modulation of the patterns inside the manifold, but not those outside (Sadtler et al., 2014). Although the conception of the neural manifold describes cell-neuron level principles of learning within a single local region (Golub et al., 2018; Chaudhuri et al., 2019; Oby et al., 2019), little is known about the constraints on the adaptation of the macroscopic sensorimotor system, that is shaped by the synchronization and desynchronization of net populational neural activities across multiple brain regions (Wander et al., 2013; Fries, 2015). Because the summation of activity of locally interconnected −10^7^ neurons cancels out the property of a single neuron and only maintains their synchronized activities which mediate information processing in the human cortical system, the principles governing the cortical adaptation processes at the macroscopic scale are putatively distinct from the local unit activities in a single region (Kelso, 2012; Tognoli and Kelso, 2014).

**Figure 1.**
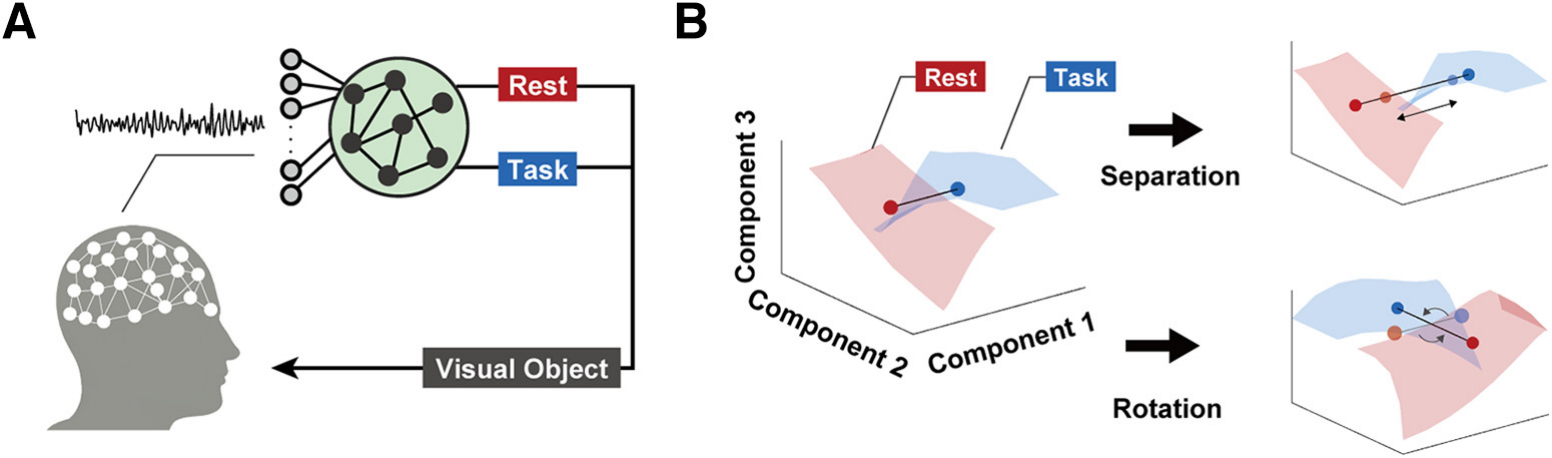
Conceptual illustration of neural adaptation process induced by brain-computer interfacing. ***A***, Setup of a brain-computer interface. Online acquired scalp electroencephalograms were fed into a classifier to detect the presence/absence of attempted movement. Predicted brain state was shown to participants as movement of visual object on display. ***B***, Conceptual visualization of cortical adaptation. Scaling adaptation reflects improvement in voluntary regulation of a specific component. If the centers of gravity determined from datapoints in two conditions are separated after brain-computer interfacing, it suggests the separability of two conditions is enhanced by adaptation. Deforming adaptation suggests that activity patterns are allocated to a specific brain state to adapt to the classifier. If the geometric relationships between two conditions are deformed with respect to a specific axis, it suggests the adaptation process progressed such that the two conditions are separated along the axis.

To investigate human adaptability at the sensorimotor network level, we used BCI operation tasks based on scalp electroencephalograms (EEG) with a variety of incorporated classifiers (Fig. 1*A*). Since users attempted to move a virtual object by exploring mental actions that effectively modulates EEG signals to control BCI, this experimental paradigm allows us to examine the relationship between BCI properties and process in the cortical adaptation (i.e., changes in neural activity patterns to fit the rule of BCI classifier). As shown in Figure 1*B*, we specifically hypothesized two distinct adaptation processes induced to improve BCI operation performance: (1) separation: rescaling of cortical activity patterns that increases geometric distances between two brain states and (2) rotation: deforming of the configuration of two brain states induced by changes in the whole-brain activity patterns. The former represents changes in the separability along with the targeted EEG feature and the latter represents rotational changes in the activity patterns toward perpendicular to the BCI classifier, respectively. The geometric analysis in the dimensionality-reduced space offers the opportunities to capture the reorganization of whole-brain neural dynamics.

To test whether the cortical adaptation process is influenced by BCI configurations, we employed three types of BCIs whose classifiers were based on different rules: model-based, *de novo* and adaptive classifiers. The model-based and *de novo* classifiers were based on fixed scalp EEG feature to induce adaptation of neural activity patterns. The model-based classifier was based on hand-area motor cortical activities and users were informed a mental strategy to successfully control BCIs, meanwhile, the *de novo* classifier was based on temporo-parietal activities and users were encouraged to explore the suitable strategy (Shibata et al., 2011). The adaptive classifier was designed to adapt to the current brain activity patterns using the whole-head EEG signals as input, facilitating the classifier-side adaptation by a block-by block calibration. We hypothesized the fixed type BCIs require reorganization of whole-brain activity patterns while the adaptive BCIs rather induce the classifier-side adaptation by intermittent calibration. Difference in the adaptation process would be characterized by geometric changes in the low-dimensional representation of neural activity patterns; BCI operation with fixed types of classifiers would lead the progress in the separation and rotation because of enhanced discriminability of brain states along with the EEG feature for classifier input. Meanwhile, the adaptive BCI would not induce those changes since the classifier-side constant calibration can optimize the classifier to fit the current brain state without changing user-side activities.

## Materials and Methods

### Participants

Twenty-one neurologically healthy adults (9 females, 12 males, mean age: 22.6 ± 3.23) naive to BCI operation participated in this experiment. The appropriate sample size for this study was determined by an a priori power analysis (α = 0.05, 1-β = 0.8, two-sided Wilcoxon signed-rank tests) focusing on the deforming effect induced by *de novo* BCI. The statistical package G*Power 3 (Faul et al., 2007) was used to estimate the sample size that shows large Cohen’s *d *=* *0.90 reported in the previous EEG-based neurofeedback literatures (Soekadar et al., 2015; Hayashi et al., 2020).

All participants had normal or corrected-to-normal vision and were asked to provide written informed consent before participating in the experiment. This study was conducted according to the ethics of the Declaration of Helsinki. The experimental protocol was approved by the ethical committee of the affiliated organization (Approval number 2020-36).

### Experimental setup

**Figure 2.**
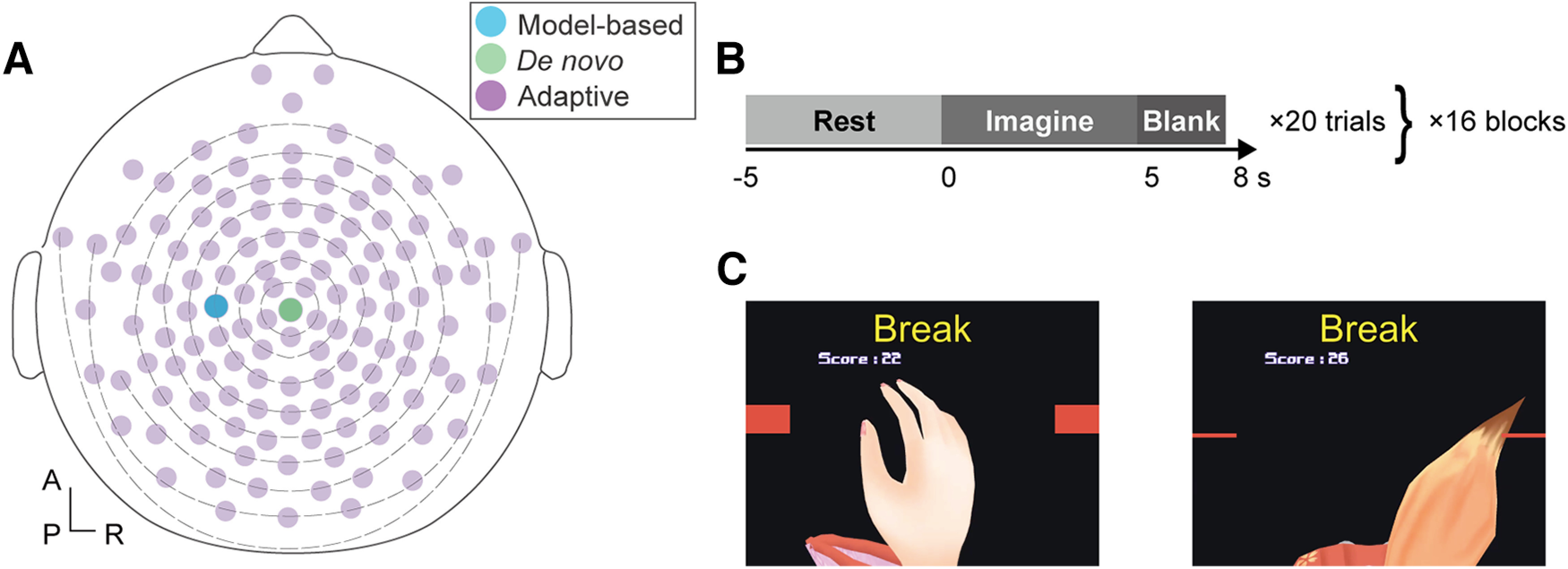
Experiment setup and protocol. ***A***, Electrode locations. The three classifiers used in the study had different channels of interest. The model-based classifier used only channel C3 indicated in blue around the left sensorimotor cortex. The adaptive classifier used whole-head EEG channels (purple) to construct a common spatial pattern. The *de novo* classifier used only the Cz channel, shown here in green. ***B***, Experimental protocol and time course of a trial. ***C***, Visual feedback object. For the model-based or adaptive classifiers, an illustration of a hand was shown that matched the attempted movements of the users while an illustration of a tail was used in the *de novo* task to encourage users to acquire novel mental actions that enhanced controllability of the BCI.

Participants were seated on a comfortable chair in a quiet room. A display was placed about one meter in front of the chair to provide task instructions and visual feedback from BCIs. EEG signals during the experiment were acquired with a 128-channel HydroCel Geodesic Sensor Net (HCGSN, EGI). The layout of channels followed the international 10–10 electrode positions shown in Figure 2*A* (Luu and Ferree, 2005). The reference channel was set to Cz. The impedance of all channels was maintained below 50 kΩ throughout the experiment. The EEG data were collected with a sampling rate of 1000 Hz.

### Experimental procedure

Participants underwent 16 BCI operation blocks comprised of 20 trials. All experimental procedures were conducted within 2 h to guarantee the reversibility of any potential effect of induced unnatural neural plasticity and investigate the initial phase of learning to operate the BCIs (Marins et al., 2019; Mehler et al., 2019; Hayashi et al., 2020). After every two blocks, participants were given a break of up to 5 min. Participants were randomly allocated to one of the three classifiers without informing the configuration of BCI, the existence of multiple types of classifiers and the allocated type of classifier was used throughout experiment (also, see below, Online processing of EEG signals).

A trial began with a 5-s “Rest” period and a 5-s “Imagine” and a 3-s “Break” period followed (Fig. 2*B*). During the “Rest” period, participants were instructed to relax without having any specific thoughts and with opened eyes. In the “Imagine” period, participants were instructed to perform motor imagery tasks based on the allocated classifiers. Participants with the model-based and adaptive classifiers imagined extending the right-hand while those with the *de novo* classifier tried moving a tail to match the attempted movement with the object on display (Fig. 2*C*). Since tail moving is not intuitive for human beings, at the beginning of the experiment, participants were encouraged to explore strategies that enables better controllability of the BCI. The strategy adopted in each block was freely determined by each participant, but they were instructed to try to use the same strategy throughout one block to acquire sufficient data and report the adopted strategy at the end of each block. Since the visual feedback for participants of the model-based and adaptive BCIs were configured to increase grasp aperture when classifier detect the motor attempt (Fig. 2*C*, left panel), they tried to keep the virtual hand opened during “Imagine” period and closed “Rest” period. Likewise, those of *de novo* BCIs were configured to move the tail toward left (Fig. 2*C*, right panel), participants tried to keep the virtual tail left side of the display during “Imagine” period and right side “Rest” period. Participants were asked not to exert overt movement during the feedback period and its compliance was visually inspected by the experimenters.

The performance of each trial was quantified by scores provided by BCI and participants were encouraged to maximize the culminative sum of score within a block. Scores were determined by the predicted presence/absence of motor attempt by classifiers. The absence of motor attempt during “Rest” periods and the presence during “Imagine” periods increased scores (reward), while the opposite prediction decreased (punishment). The changing rates of these scores were pertinent to the metrics used for feedback by each classifier and were regulated linearly to fit the score range from −100 to 100. For the adaptive classifier, the common spatial pattern (CSP)-support vector machine (SVM) model was trained with data from the previous block and the trained model was used in the next block. Note that the first block of users allocated to the adaptive classifier was identical to that of the model-based, to collect a dataset for the adaptive classifier training.

### Online processing of EEG signals

To test the initial adaptation process during BCI use, we prepared three types of binary EEG classifier that detects presence of human motor attempt from based on different EEG features. The following processing was conducted using MATLAB R2019a (The MathWorks, Inc.) and Unity (version 2019.2.4f1, Unity Technologies). Online acquired EEG signals were processed with a 1651-point, minimum-phase, FIR 8- to 30-Hz bandpass temporal filter and then processed with one of the three types of BCI classifiers. Online processed EEG signals were used to detect the presence of motor attempt with one of the three types of classifiers: model-based, adaptive, or *de novo*. Each classifier was designed with different rules, and electrodes of interest were defined as shown in Figure 2*A*. During experiment, users were instructed to use one of three BCIs at the time course defined as Figure 2*B* (also, see above, Experimental procedure).

The model-based classifier was constructed based on those used in sensorimotor rhythm (SMR) BCIs (Buch et al., 2008; Kraus et al., 2016). Because accumulated evidence suggests that event-related desynchronization of SMR (SMR-ERD) contralateral to the hand that attempted to move reflects the excitability of SM1 (Hummel et al., 2002; Takemi et al., 2013; Naros et al., 2020), EEG signals around the left SM1 (i.e., channel C3) were only used to detect the attempted movement. In online processing, a large Laplacian filter was applied to EEG signals from channel C3 to extract sensorimotor activity (McFarland et al., 1997; Tsuchimoto et al., 2021). Subsequently, the band power of SMR (SMR-power; 8–13 Hz) was extracted by Fourier transform with a 1-s window and Hamming window function. The magnitude of SMR-ERD (dB) was computed from the obtained SMR-power with the following formula:

ERD(t)=−10 log10(P(t)/PRef),

where P(t) denotes the signal power of EEG signal at the channel and frequency of interest, here the SMR-power, at time point t, and ${\text{P}}_{Ref}$ denotes the reference power (Pfurtscheller and Lopes Da Silva, 1999). The reference power ${\text{P}}_{Ref}$ was calculated from the middle 3-s period of “Rest” time from the previous trial. Note that the ERD values were determined independent from classifier parameters. During BCI operation based on the model-based classifier, movements of the illustrated hand in the display and performance scores were defined to be linearly related to the SMR-ERD value in the range of 0–10 dB (Fig. 2*C*, left panel). The range grasp aperture was discretized to 100 steps (0 dB: fully closed, 10 dB: fully opened) and scores were calculated by the integral of SMR-ERD. One may point out the necessity of user-specific model calibration to identify responsive frequency or channels of interest. However, we used the identical classifier across participants to avoid the potential confound that the effectiveness of calibration interacts with learning efficacy.

The *de novo* classifier had a fixed classifier plane as did the model-based classifier, however, its characteristics were biologically unnatural; the *de novo* classifier was based on EEG signals around the temporo-parietal region (i.e., channel Cz) that are associated with not only sensorimotor, but also attentional features (Benedek et al., 2014; Misselhorn et al., 2019). Actively exploring suitable mental strategies, users attempted to move their body or a visual object on the display during the BCI task. However, the motor imagery of corresponding body parts at the region (i.e., foot) and increased attention do not contribute to the spectral power attenuation in the α band (8–13 Hz) required by the classifier. Specifically, since the α band power was increased by the motor attempt of moving the feet or by internal attention at the targeted channel (Pfurtscheller et al., 2006; Benedek et al., 2014), such intrinsic responses did not contribute to the BCI operation, Online computed ERD magnitude with the procedure identical to that from channel C3 in the model-based classifier was exploited to decode the absence/presence of attempted movement and index for neurofeedback. The angle of tail was discretized to 100 steps (0 dB: right limit, 10 dB: left limit). Note that the rules for object movement were identical to those of the model-based classifier.

Lastly, the adaptive classifier was constructed using whole-head scalp EEG signals based on a common spatial pattern (CSP) algorithm and a support vector machine (SVM; Pfurtscheller et al., 2006; Blankertz et al., 2007). To adapt to the current activity patterns of users, CSP components that maximize the separability of the two conditions “Rest” and “Imagine” were trained at the end of each block. SVM classifiers were constructed to perform a binary classification of the two conditions based on 6 CSP components. Although the CSP-based feature extraction did not employ time-frequency transformation for spectral power calculation used in the model-based and *de novo*, users of adaptive BCIs were also required to perform kinesthetic motor imagery which modulates spectral power of scalp EEG. The posterior probability for a data point classified as presence of motor attempt was used as an index for neurofeedback; the index for the adaptive classifier was defined to be linearly related to the posterior probability in the range of 50% to 100%. Note that the rules for object movement and for obtaining scores were identical to those in the other two types of classifiers.

### Evaluation of BCI performance

For each participant, online-calculated scores were individually subjected to linear regression analysis to summarize whether performance of participant improved over blocks for each classifier (Gruzelier, 2014; Kober et al., 2018; Witte et al., 2018). The score obtained during a given block was used as a dependent variable and block number was used as a predictor valuable. If scores increased during the experiment, the regression coefficient for the predictor valuable was positive. After the regression coefficients were derived from scores of each participant, they were subjected to a group-by-group Wilcoxon rank-sum test with a false discovery rate correction to test whether the obtained regression coefficients were significantly different from zero (Benjamini–Hochberg method; Benjamini and Hochberg, 1995). If significant positive shift of the slopes were observed, the result indicated systematic progress of controllability improvement for the BCI. However, note that the comparison of the learning rate across groups are not applicable because of the difference in score calculation procedure. Moreover, to capture the difference in performance at the beginning and end of experiment, acquired scores were compared with Wilcoxon signed-rank test for first and last four blocks of each BCI operation (early and late period, respectively).

### Offline EEG preprocess

The recorded EEG signals were first preprocessed with EEGLAB (Delorme and Makeig, 2004) to reject artifacts and enhance the computational efficiency with downsampling (Bigdely-Shamlo et al., 2015) The raw EEG data were filtered with a zero-phase 1–45 Hz FIR bandpass filter, downsampled to 100 Hz and bad channels identified by clean raw data plugin were removed from further analysis. The removed channels were interpolated spherically to minimize a potential bias when re-referencing the electrodes to a common average reference. Subsequently, large-amplitude artifacts caused by blinking or head displacement were removed with artifact subspace reconstruction algorithm (Kothe and Makeig, 2013). The electrodes were then re-referenced to the common average reference to extract activity specific to the electrodes (McFarland et al., 1997).

The continuous EEG data were then segmented into trials to evaluate the middle 8-s periods of the online BCI training trials (i.e., the last 4 s of the “Rest” period and the first 4 s of the “Imagine” period). To obtain the independent EEG components of the segmented dataset, we used adaptive mixture independent component analysis (AMICA; Palmer et al., 2011). Finally, an automatic artifact rejection was applied using ICLabel that distinguished genuine EEG components from artifacts induced by eye, muscle, heart, line noise, and channel noises (Pion-Tonachini et al., 2019).

To investigate cortical adaptation processes during brain-computer interfacing, the band-power features were used as a raw-vector that represents instantaneous overall brain state. Computed band-power from each EEG channel was subdivided into five functionally distinct frequency bands (δ: 1–4 Hz, θ: 4–8 Hz, α: 8–13 Hz, β: 13–31 Hz, γ: 31–45 Hz; Hayashi et al., 2020). The averaged band-power was log-transformed and normalized to the *z* score in a trial-by-trial manner to cancel baseline drifting. Thereby, the original number of dimensions of the feature vector D was D=129×5=645. Note that the feature targeted by the model-based and *de novo* classifiers were included in *D*.

### Feature extraction of EEG-dataset using t-distributed stochastic neighbor embedding (t-SNE) algorithm

**Figure 3.**
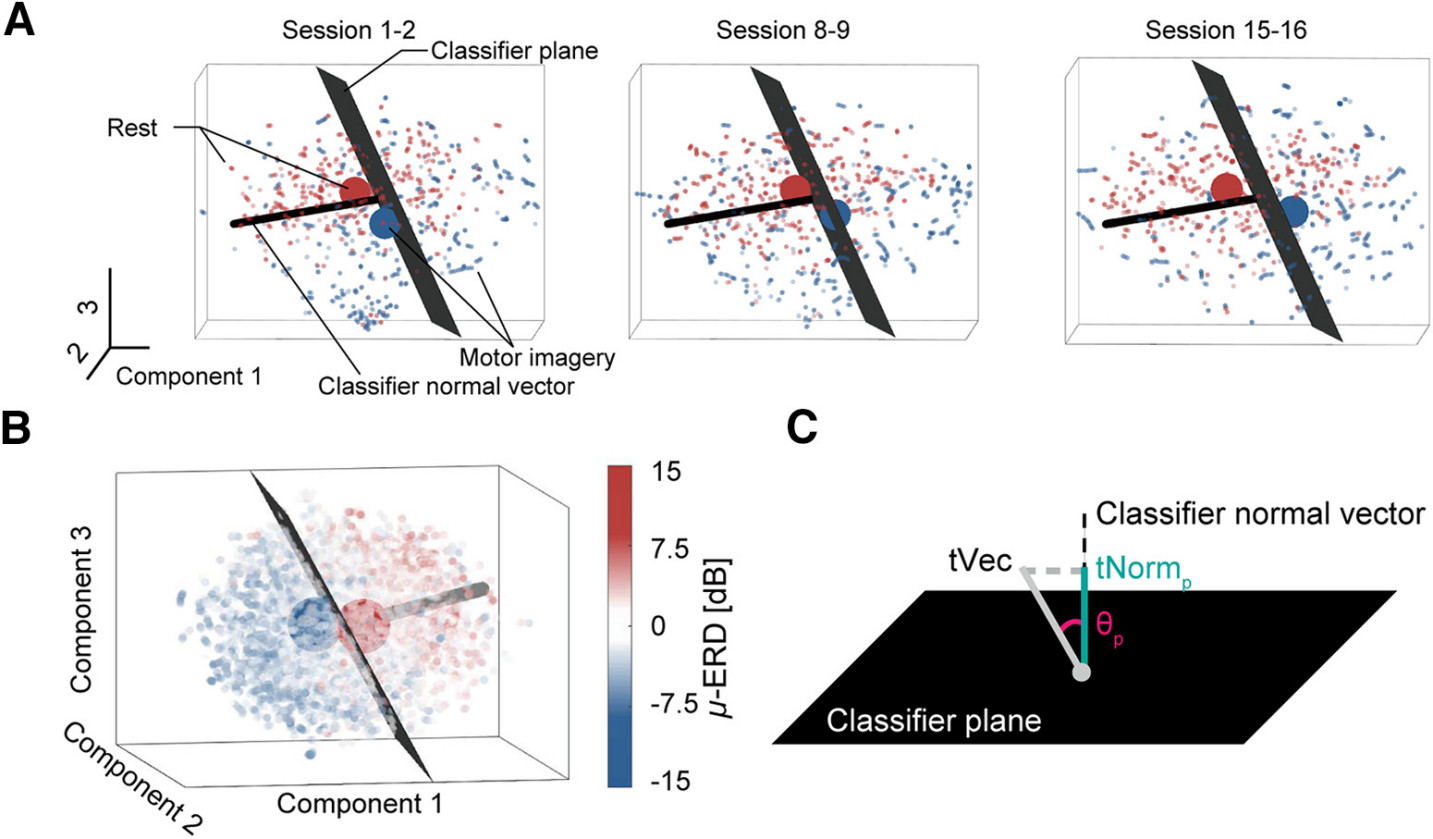
Low dimensional visualization of EEG data by t-SNE. ***A***, Changes in geometric relationships between dataset and classifier plane. As training progressed, the geometric relationship of points from two brain states changed with respect to the classifier plane (black plane). The large points indicate the centers of gravity of points from each brain state. The black line orthogonal to the classifier plane is the classifier normal vector (see also Figure 3*C*). ***B***, An example of t-SNE-based data visualization in embedded space (Model-based classifier user). Each datapoint is colored with its SMR-ERD value derived from the C3 electrode around the left sensorimotor cortex. The black plane represents the classifier plane (see also Classifier plane and geometric assessment of EEG data for mathematical details). The large points indicate the centers of gravity of points from each brain state. The black line orthogonal to the classifier plane is the classifier normal vector (see also Figure 3*C*). ***C***, The t-SNE-based quantification of the adaptation process with respect to the classifier plane. *tNorm_p_* is defined as a component of *tVec* with respect to the classifier vector, while θ*_p_* is defined as a subtended angle between *tVec* and the classifier vector.

The preprocessed EEG dataset (645 × 11,520 matrix) was subjected to a subject-by-subject t-SNE analysis, which converted the pairwise distances between data points in the original feature space to conditional probabilities (Van Der Maaten and Hinton, 2008). The t-SNE algorithm minimized the Kullback–Leibler divergence representing the distance between the conditional probability in the original and embedded space, where conditional probability that the data points $x_{i}$ and $x_{j}$ are neighbors was calculated from the pairwise distances of input data. In this study, the number of dimensions of EEG features was reduced to three with a Barnes-Hut variation of t-SNE (Van Der Maaten et al., 2014) to speed up the computation. Perplexity, that is a hyperparameter of the t-SNE algorithm, was set to 20 determined empirically with a parameter search of past EEG data for best separation between the “Rest” and “Imagine” periods. The hyperparameter was fixed across participants throughout the study after the determination. After applying t-SNE, the dimensionality-reduced datasets were subjected to visualization and a similarity analysis, but classification labels (i.e., “Rest” or “Imagine”) were determined from the original dataset (Fig. 3*A*).

### The t-SNE-based dimensionality reduction and quantitative analysis in embedded space

Feature extraction using dimensionality reduction is popularly conducted for high-dimensional neural data across modalities (Cunningham and Yu, 2014; Lord et al., 2019). The t-SNE algorithm we employed for dimensionality reduction is advantageous for geometric evaluation as it preserves original distances in the embedded space. Because t-SNE unfolds the nonlinear structure of a given dataset, the linear distance in the embedded space can be interpreted as an approximation of geometric distance in the original space. It illustrates how different one brain activity pattern is from another; however, it should be noted that to properly interpret the results, (1) distance scales in the embedded space were rearranged and were variable across iterations of t-SNE, (2) distance scales in different clusters might have differed, and (3) direct comparisons of distances between clusters were not acceptable because distances within two clusters were arbitrary. To deal with the above concerns, two approaches were adopted: (1) data points were bridged to prevent the formation of multiple clusters, and (2) statistical distances, namely, Hotelling’s t-squared statistical values, were used instead of Euclidean metrics. Because distances between nearby points are well preserved in embedded space, the distance scale of distant points were kept similar for enough data points, which acts as a bridge and prevents the formation of sparse multiple clusters. We also adopted the concept of “short-circuiting” (Lee and Verleysen, 2005) by constructing the feature vectors with overlapped time-windows so that points were smoothly connected, and all data acquired from single participants were subjected to t-SNE algorithm at once. Thus, distances from point to point shared the same scale across all points (i.e., only one cluster was generated in embedded space as shown in Fig. 3*B*).

Hotelling’s t-squared statistic was adopted as the distance metrics between two group of points (Hotelling, 1992). Assume x and y are two groups of points lying in a *p*-dimensional space, $n_{x}$ and $n_{y}$ are the numbers of points, $\bar{x}$ and $\bar{y}$ are the sample means, and ${\hat{\Sigma }}_{x}$ and ${\hat{\Sigma }}_{y}$ are the respective sample covariance matrices. The Hotelling’s t-squared statistic was calculated as:

$$t^{2}=\frac{n_{x}n_{y}}{n_{x}+n_{y}}{\left(\bar{x}-\bar{y}\right)}^{\text{′}}{\hat{\Sigma }}^{-1}(\bar{x}-\bar{y})$$

$$\hat{\Sigma }=\frac{\left(n_{x}-1\right){\hat{\Sigma }}_{x}+\left(n_{y}-1\right){\hat{\Sigma }}_{y}}{n_{x}+n_{y}-2}.$$

Hotelling’s t-squared statistic is suitable for measurements of statistical distance in the t-SNE-embedded space, as they were invariant to the distance scale. The distribution of $t^{2}$ follows an *F*-distribution:

$$t^{2}∼\frac{p\left(n_{x}+n_{y}-2\right)}{n_{x}+n_{y}-p-1}F_{p,n_{x}+n_{y}-1-p}.$$

To normalize the distribution, the square root of $t^{2}$ was defined as tNorm and was used as the distance measurement in subsequent analyses:

$$t\text{Norm}=\sqrt{t^{2}}.$$

The vector representing the directional relationship between two classes was defined as a 3D vector tVec:

$$tVec=t\text{Norm}\cdot \frac{\bar{x}-\bar{y}}{\bar{x}-\bar{y}}.$$

Data points were divided into two classes: “Rest” and “Imagine” according to their relative times in the trials. tNorm and tVec were calculated for these two conditions.

### Classifier plane and geometric assessment of EEG data

To investigate the influence of BCI classifiers on the cortical adaptation in the t-SNE-embedded space, the classifier plane and classifier normal vector were linearly projected into the embedded space (see Fig. 3*C*). A 3D classifier normal vector $V={\left[v_{1},v_{2},v_{3}\right]}^{T}$ was calculated as follows, where T denotes a matrix transpose:

$$X=\left(\begin{array}{c} 1\\ ⫶\\ 1 \end{array}\;Y\right),\;\left(\begin{array}{c} b\\ \vec{v} \end{array}\right)={\left({\text{X}}^{T}X\right)}^{-1}X^{T}P$$

$$V=\vec{v}/\vec{v}.$$

Then, the equation of the classifier plane is given as follows:

$$v_{1}x+v_{2}y+v_{3}z+b=0,$$

assuming $Y\in {\mathbb{R}}^{N\times 3}$ are the points in the 3D embedded space (three dimensions were represented as x,y,z, respectively), $P\in R^{\left(N\text{×}1\right)}$ are the original features referred to by the classifier (model-based: α-ERD at C3, *de novo*: α-ERD at Cz, adaptive: classifier score), where *N* is the number of points (11,520), b is the intercept corresponding to the decision boundary of the classifiers. The classifier normal vector was derived using the ordinary least squares by minimizing the error between the value of the feature and those estimated from the coordination in the low-dimensional space. As is shown in Figure 3*C*, tVec were projected to the classifier normal vector to evaluate its geometric relationship against the classifier. The lengths of projection on the classifier vector ( $t{\text{Norm}}_{\text{p}}$) and the angles between tVec and the classifier vector ( $\theta_{p}$) were calculated across classifiers as follows:

$$t{\text{Norm}}_{\text{p}}=tVec\cdot V$$

$$\theta_{p}=\arccos\frac{tVec\cdot V}{tVec}.$$

Because $t{\text{Norm}}_{\text{p}}$ reflects the size of component in tVec aligned with the classifier normal vector, the increase in the $t{\text{Norm}}_{\text{p}}$ indicates how two brain states are separated by the classifier. Meanwhile, $\theta_{p}$ indicates how the relative position of the two states is aligned with the classifier normal vector.

### Geometry-based analysis in the embedded space

The geometry-based analysis was conducted in the embedded space, as geometric relationships of the points reflected the similarities in the original space. The transition process from one brain condition to another (i.e., absence to presence of attempted movement) was assessed by the spatial arrangement and separability of points from the “Rest” and “Imagine” periods in the t-SNE dimension (Fig. 1). Emergence of the two temporal phenomena were defined as follows:

- Separation: The separability of the two conditions (Rest and Imagine) increases with respect to a fixed axis. Separation is interpreted as the enhancement of specific cortical activity patterns.
- Rotation: The relationship of positions in the two conditions changes direction. Deforming is interpreted as an alteration of a cortical activity pattern that is adopted as the rotational changes towards perpendicular to the classifier plane indicates the reconfiguration of activity patterns contributing to BCI performance improvement.

To quantify the two distinct adaptation process, the following metrics were defined. Scaling and deforming between the ith and jth blocks were, respectively, quantified by the difference of $tNorm_{p}$ and $\theta_{p}$.

If adaptation progresses toward the targeted neural activity patterns required to control BCIs, the $t{\text{Norm}}_{\text{p}}$ values should be larger while those of $\theta_{p}$ should be smaller. Thus, the calculated values were subjected to the Wilcoxon signed-rank test to compare the differences between the early and late periods. For adaptive classifiers, as the classifier plane was obtained from the second block, we defined early period as two to five blocks for the classifier and the classifier normal vector was approximated by the mean of vectors derived from trained with the previous blocks. We then corrected the α-level with a Bonferroni correction.

### Cortical source estimation

To localize the source of neural signaling during BCI operation, EEG signals were subjected to sLORETA analysis for cortical source estimation (Pascual-Marqui, 2002). Because the motivation for conducting the source analysis was to test whether the targeted region of the classifier was successfully activated during the late period of BCI training, averaged data from early and late periods were subjected to a nonparametric permutation test (Nichols and Holmes, 2002).

## Results

### Participants learnt BCI operation based on the mental actions

**Figure 4.**
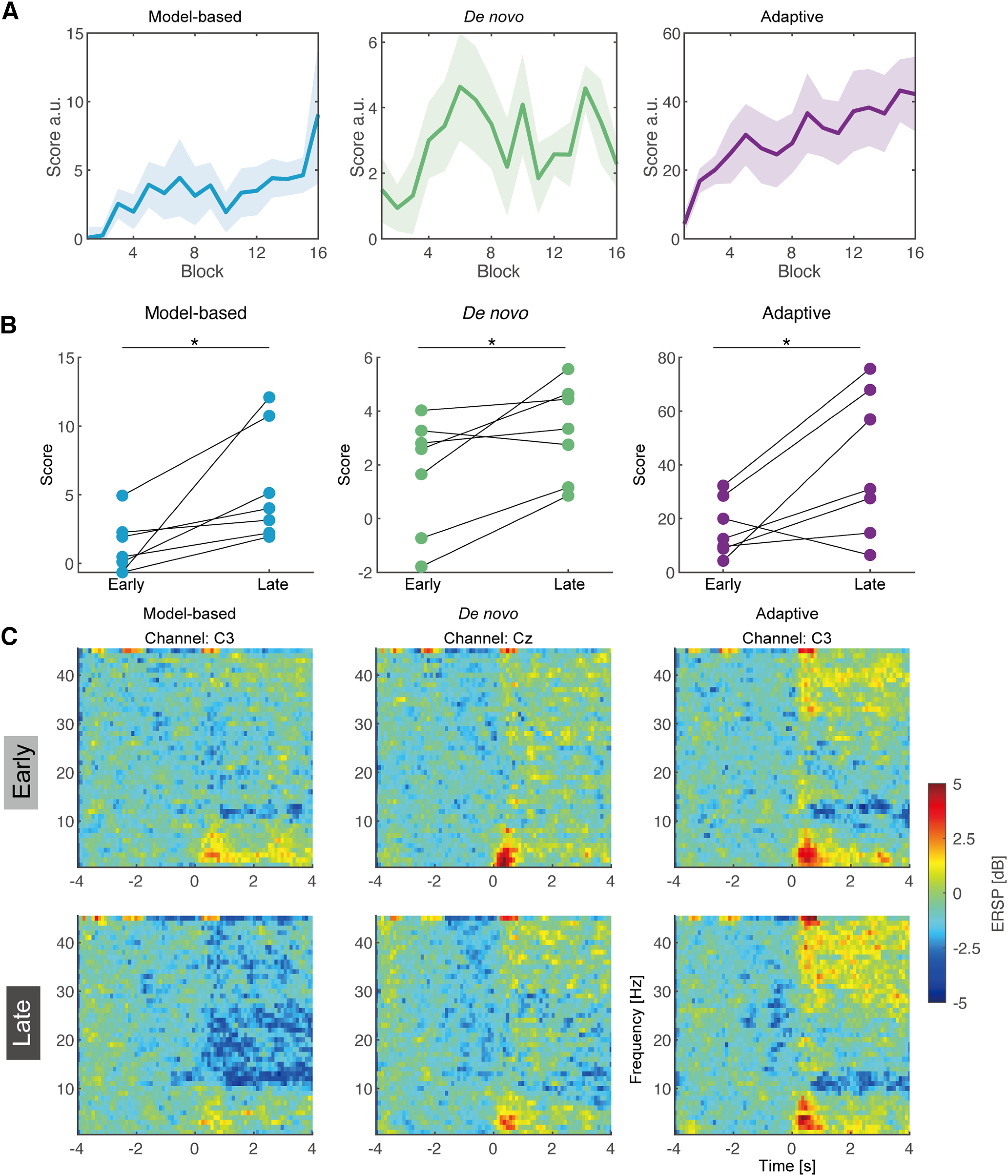
Changes in BCI operation performance and time-frequency representations of scalp electroencephalogram signals. ***A***, Group results of performance scores from users of model-based, *de novo* and adaptive classifiers. Solid lines indicate mean values while shaded areas represent 1 SE across participants. ***B***, Changes in the acquired scores during BCI operation. Asterisks indicate statistical significance (*p* < 0.05). ***C***, Changes in time-frequency representations of scalp electroencephalogram signals from representative channels.

**Figure 5.**
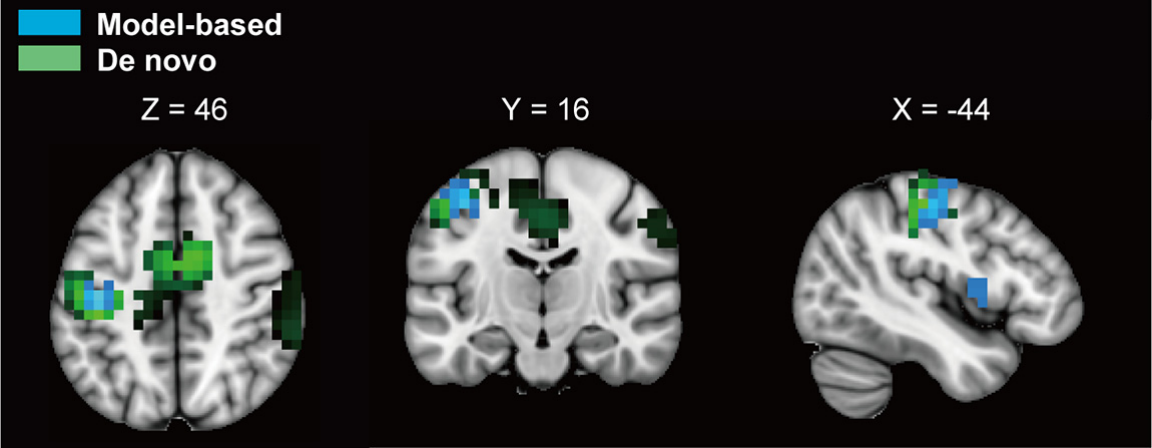
Spatial activity patterns during brain-computer interfacing. Results of source estimation analysis from representative participants. The colored regions indicate voxels where activities were significantly different during Rest and Imagine periods (*p *<* *0.05 unc.). Areas colored with blue and green indicate those for model-based and *de novo* classifiers, respectively. While significant voxels were localized around the contralateral hemisphere of the imagined hand for the model-based classifier, those for the *de novo* classifier were located bilaterally, including in the pre/postcentral gyrus and supplementary motor area (peak voxel was in the postcentral gyrus; MNI coordinates: −40, −25, 45). Note that a representative source estimation for the adaptive classifier is not shown because of variable activity patterns among participants. sLoreta analyses of statistical nonparametric mapping for estimated cortical sources of band power in the α band (8–13 Hz). Areas colored with blue and green indicate those from model-based and *de novo* classifiers, respectively. Masks superimposed on a standard brain template were visualized by MRIcroGL (https://www.mccauslandcenter.sc.edu/mricrogl/home).

Twenty-one participants operated BCIs with one of three randomly allocated classifiers that provided scores contingent on BCI. Since culminated sum of scores in a block represents the overall performance of BCI operation, we tested whether the performance improvement was systematically observed in participants of each BCI (Fig. 4*A*). While BCI performance scores from the model-based and adaptive classifier generally increased over blocks, those for the *de novo* classifier did not. Regression coefficients of linear regression analysis were computed based on acquired scores from each participant, using the block numbers as the explanatory variable and the acquired scores as response variables. Statistical tests to test for computed regression coefficients revealed significant differences from zero for BCIs based on the model-based and adaptive classifiers (model-based: *p *=* *0.0078, *d *=* *1.86, adaptive: *p *=* *0.023, *d *=* *0.97, *de novo*: *p *=* *0.055, *d *=* *0.74, Wilcoxon rank-sum test, FDR corrected). The comparison of acquired scores at early and late period indicate significant difference across groups (Wilcoxon signed-rank test, all *p* < 0.05; Fig. 4*B*), indicating even *de novo* BCI elicits adaptation of participants through training. Note that direct comparison of the coefficients among classifiers is not possible because scores from each classifier were computed based on different EEG features (Figs. 4*C*, 5). As shown in Figure 4*C*, the time-frequency representations of scalp EEG signals derived from channels of interest for the fixed classifiers (i.e., C3 channel for model-based and Cz for *de novo*), qualitatively exhibited the changes in the SMR-ERD magnitude during motor imagery (from 0 to 4 s) from early to late period of BCI operation training. For the fixed classifiers using a predefined feature for the motor attempt detection, the spatial representation was visualized by the cortical source estimation (Fig. 5). The corresponding features exhibited sensorimotor activity corresponding to the feature on interest (i.e., model-based classifier: activity around contralateral SM1, *de novo* classifier: activity around temporo-parietal region).

### Geometric quantification of cortical adaptation process revealed distinct adaptation processes to classifier’s separating plane

BCI training requires users to control the voluntary control of targeted activity which classifiers use for motor attempt detection. However, not only the targeted features, those derived from regions interconnected with the target would also reorganize through learning (Wander et al., 2013; Corsi et al., 2020). To examine differences in cortical adaptation processes, we investigated changes in whole-head EEG signals for the early and late period (first and last four blocks of BCI operation, respectively). An example of data from the model-based classifier BCI is shown in Figure 3*A*. As the participant performed the BCI operation, data during attempted movement (blue points) moved across the classifier plane, where the sign of relative SMR power flips (Fig. 3*B*). In this case, the defined metrics $tNorm_{p}$ and $\theta_{p}$ (Fig. 3*C*), respectively, increased and decreased. The classifier normal vector used to calculate those metrics exhibited statistical significance across blocks and indicated comparable $R^{2}$ values across BCI types (Model-based: $R^{2}$ = 0.23 ± 0.1, *de novo*: $R^{2}$ = 0.26 ± 0.2, Adaptive: $R^{2}$ = 0.21 ± 0.2).

**Figure 6.**
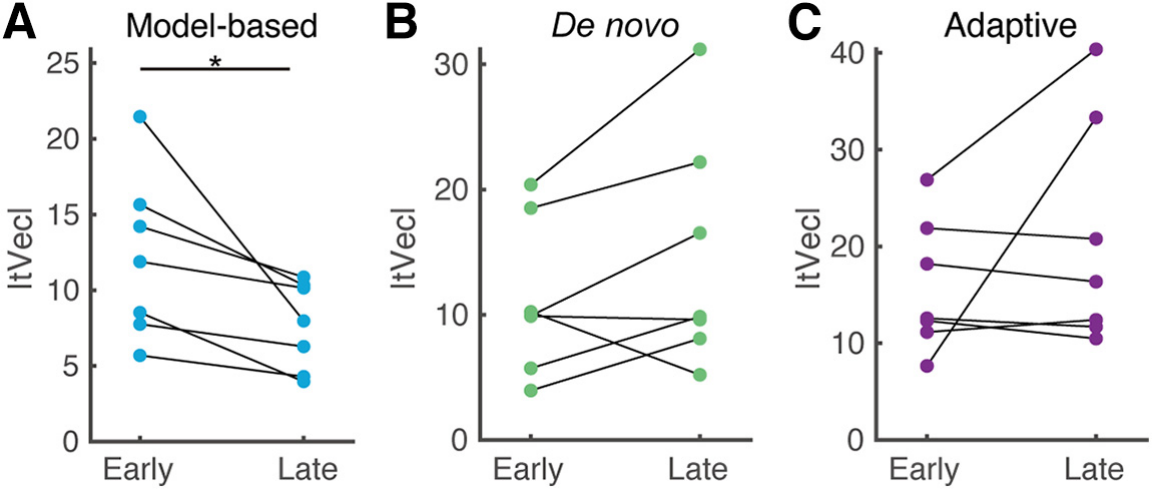
Overall changes in distance between brain states. Changes over time in the norm of *tVec* for participants operating under the model-based classifier (***A***), the *de novo* classifier (***B***), and the adaptive classifier (***C***). Asterisks indicate statistical significance (*p* < 0.05).

**Figure 7.**
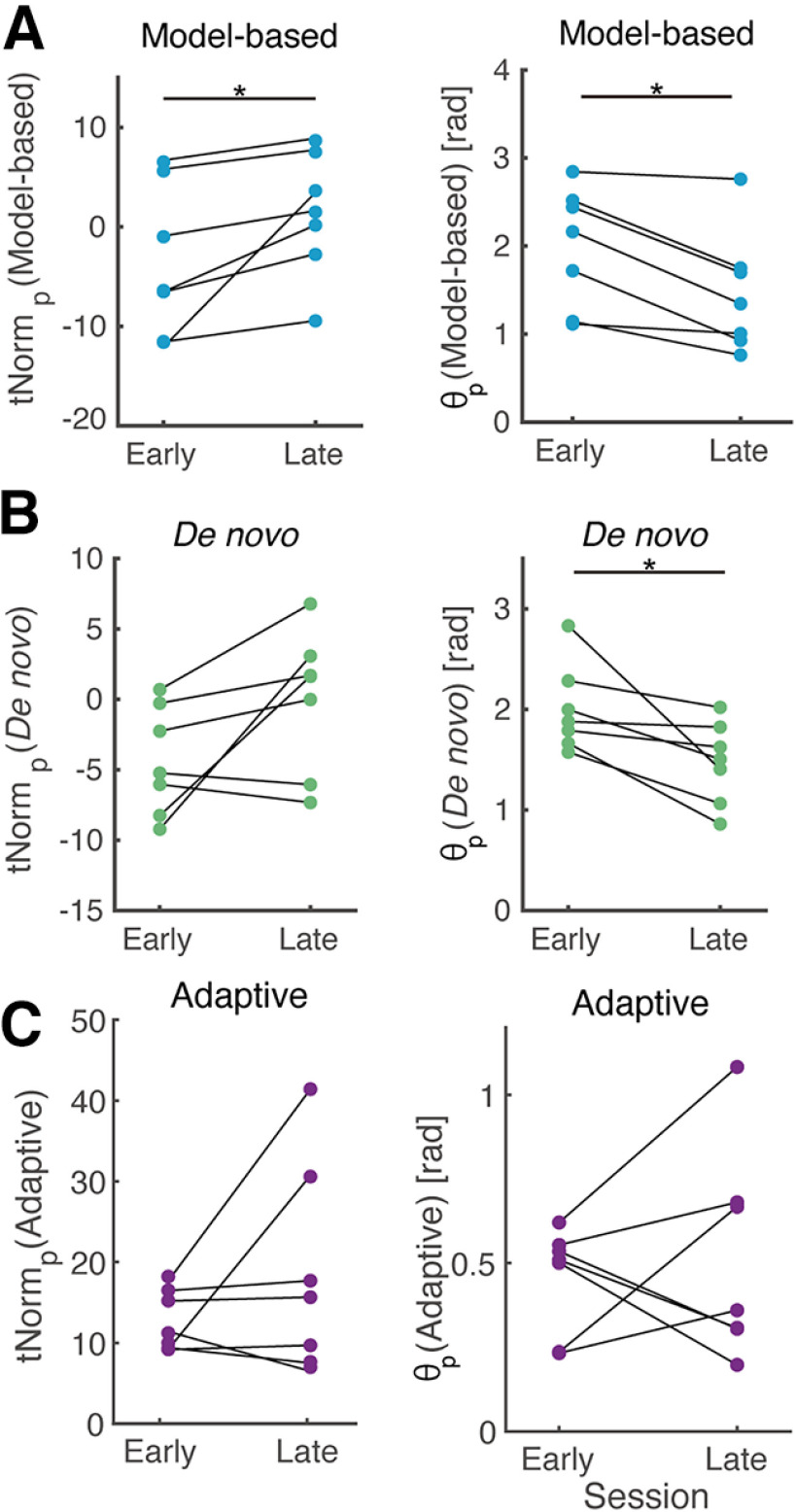
Quantitative comparison of cortical adaptation processes in embedded. Changes over time in $tNorm_{p}$ and $\theta_{p}$ for participants operating under the model-based classifier (***A***), the *de novo* classifier (***B***), and the adaptive classifier (***C***). Asterisks indicate statistical significance (*p* < 0.05).

Figure 6 indicates changes in the norm of tVec(|tVec|) between early and late period. Because |tVec| is determined by the distance between the averaged points of two brain states, it change reflects the overall activity changes including the modulation of EEG component irrelevant to BCI control. For participants trained with model-based classifier |tVec| significantly decreased (*p *=* *0.016, *d *=* *1.02, two-tailed Wilcoxon signed-rank test), while no systematic changes were observed for other two types (*de novo*: *p *=* *0.22, adaptive: *p *=* *0.81), suggesting the whole-brain activation patterns did not exhibit increased separability in any of three BCIs. However, despite the decrease in the overall norm of tVec, $tNorm_{p}$ values, the component of *tVec* relevant to the EEG component used for the motor attempt detection by the classifier (i.e., ERD in α band at contralateral SM1) significantly increased in the participants of the model-based BCI (Fig. 7*A*, *p *=* *0.016, *d *=* *0.71). At the same time, $\theta_{p}$ values decreased significantly for participants trained with both the model-based (*p *=* *0.016, *d = *0.77), indicating the reorganization of whole-brain activity patterns toward perpendicular to the classifier plane. Note that the negative values of $tNorm_{p}$ observed in some participants of model-based and *de novo* BCIs are because of the use of fixed classifier normal vectors derived from whole-experiment data including unsuccessful BCI control.

The identical evaluation was conducted for the *de novo* classifiers. Figure 7*B* depicts changes in $tNorm_{p}$ and $\theta_{p}$ against the *de novo* classifier. While no significant differences were confirmed for $tNorm_{p}$ values over blocks (*p *=* *0.078), $\theta_{p}$ values significantly decreased (*p *=* *0.016, *d *=* *1.3), suggesting the partial adaptation to the classifier plane requiring biologically unnatural EEG responses through exploration (i.e., ERD in α band at temporo-parietal region).

As the classifier planes changed from one block to the next for the adaptive classifiers trained with the data from the previous blocks, each metric was calculated against the classifier plane determined with the dataset from the previous block. No significant differences in $tNorm_{p}$ or $\theta_{p}$ were confirmed for comparison between the early and late period for the adaptive classifiers (Fig. 7*C*, *p *=* *0.47, *p *= 0.82, respectively). Since the analysis on predetermined sample size detects statistically significant changes if five out of seven participants exhibit systematic changes, the result suggests no evidence in the adaptation of neural activity patterns was found for the adaptive classifier recalibrated at the end of each block.

In summary, short-term BCI operation training elicited different cortical adaptation processes depending on the BCI types; the model-based and adaptive classifier elicited group-level systematic learning while *de novo* did not. Meanwhile the two fixed classifiers induced adaptation of neural activity patterns to improve BCI operation performance by reorganizing the whole-brain cortical activity patterns evaluated in the t-SNE space. Further, the performance improvement elicited by the adaptive classifier was mainly driven by the classifier-side adaptation rather than the cortical adaptation as suggested by the no evidence of changes in any of metrics of neural activity patterns.

## Discussion

In the present study, participants performed BCI operations with one of three classifiers: model-based, adaptive, or *de novo*. Because BCI paradigm allows experimenters to set an arbitrary relationship between the BCI model and users (Sadtler et al., 2014), changes in acquired scores are fully attributed to changes in the targeted feature. Although learning curve of acquired scores indicated model-based and adaptive classifiers exhibited significant improvement for BCI control, the adaptation processes were likely distinct. Each classifier elicited a different cortical adaptation process consistent with their characteristics; for the model-based classifier the t-SNE analyses in embedded space revealed decrease in *|* tVec| and increases in $tNorm_{p}$ that is the metric for separation of the neural manifold with respect to the axes orthogonal to the fixed decision boundary. Meanwhile, for the adaptive classifiers, changes in populational activities were not induced. Lastly, decrease in $\theta_{p}$, that is the metric for deforming effect reflecting reconfiguration of neural manifold orthogonal to its classifier plane, was induced by the *de novo* classifier based on biologically unnatural features. Because the present study focused on the difference in the performance improvement of BCI control at the initial stage, binary classifiers employed in the three types of BCIs were putatively suitable for the naive BCI users to learn its control within short-term period. The findings would also contribute to the adaptation process to the BCI with multivariate classifiers whose performance is improved through gradual increase in degree-of-freedom (Benabid et al., 2019; Edelman et al., 2019).

Users of model-based BCI demonstrated overall improvement of acquired scores and the increase in $tNorm_{p}$. Because $tNorm_{p}$ indicates increase in the separability of the two states to improve performance of BCI operation, its increase suggests scaling effect along the axis orthogonal to the decision boundary. The systematic increase in separability was only observed for the model-based classifiers that required the attenuation of SMR derived from contralateral hemisphere to imagined hand while the model-based BCI induced decrease in the absolute length of tVec. The contradictory changes, that decrease in overall norm and increase in norm of the projection to decoder normal vector, would be explained by the suppression of signaling changes not beneficial for BCI operation since the model-based BCI determines the presence of motor attempt only based on the electrode from targeted region (i.e., contralateral SM1). Such selective modulation of specific component is consistent with motor skill acquisition (Bassett et al., 2015) as well as previous reports of adaptation of neural activity patterns during BCI operation (Corsi et al., 2020; Hennig et al., 2021). Collectively, the scaling effect evaluated by $tNorm_{p}$ would be mainly driven by the selection of activity patterns rather than the emergence of new patterns which elongates the manifold. The finding about reorganization process of whole-brain activation patterns, that is concurrent improvement of modulating task-relevant and suppressing task- irreverent activities in the early phase of BCI training, is consistent with those observed for motor learning (Dal’Bello and Izawa, 2021), suggesting utility of the geometric assessment to evaluate adaptation process. Because the t-SNE analysis employed in the present study focused on the adaptation along with the axis perpendicular to the classifier plane, the process of the autonomation of cortical activities was not fully investigated in the present study. The more specialized investigation based on the present finding would be warranted using a large cohort of populations experiencing BCI operation with the combination of customization of BCI classifier to fit user-specific SM1 activities (e.g., frequency and channel selection based on calibration data).

Another fixed classifier, namely, *de novo* classifier did not elicit the systematic changes in the $tNorm_{p}$. Although the EEG feature the *de novo* classifier used was derived from the temporo-parietal α activity, participants did not exhibit systematic adaptation observed in those of the model-based classifier. This difference might have stemmed from not only the classifier configuration, but also instruction about the task. Since exploration behavior was encouraged during *de novo* classifier operation, the instruction may lead association of the specific patterns with better control of BCI and acquisition of covert mental strategies in an implicit manner through neurofeedback (Shibata et al., 2011). As motor tasks adapted through such an exploratory strategy might require more extensive training than recalibrating the existing control configuration (Radhakrishnan et al., 2008; Telgen et al., 2014; Choi et al., 2020), multiday training of the *de novo* BCI operation would induce the sophistication of BCI operation by adopting exploration strategy (Fujisawa et al., 2019).

Rotational effect was quantified by another metric for geometric evaluation, $\theta_{p}$ that indicates the angle between classifier normal vector and tVec. While the increase in $tNorm_{p}$ indicated two brain states became more separable with respect to the features used in classifiers, the decrease in the $\theta_{p}$ indicated the changes in cortical activity patterns during the BCI operation. Significant changes in $\theta_{p}$ were observed for not only for model-based classifiers but also *de novo.* Although the absence of increase in $tNorm_{p}$ was concomitant with that of obtained scores dependent on the targeted EEG feature, the cortical adaptation that partly contributed to th*e de novo* BCI operation was probed by $\theta_{p}$ changes. As the $\theta_{p}$ is the nonlinearly related to tVec (the overall distance between the two brain states), the metric is more sensitive to changes in the geometric configuration than $tNorm_{p}$ which is linear function of tVec (see Materials and Methods, Classifier plane and geometric assessment of EEG data).

One potential limitation of the t-SNE analysis employed in the present study is that the variance of features explained by the classifier vector becomes relatively low because of the nature of dimensionality reduction algorithms. Since the high-dimensional brain activity patterns (645 dimensions) were compressed into 3D spaces, the explained variance of features by classifier normal vectors became overall 20%. Nevertheless, because we observed consistent statistical significance of linear regression models across participants and the preserved variance was sufficient to detect the reorganization process along with the features targeted by BCIs in keeping with the univariate analyses on acquired scores during BCI operation, the estimated classifier normal vectors were statistically reliable representation of features targeted by BCIs. Given that t-SNE algorithm preserves relative distances of each data point in the original space, we believe the t-SNE analysis would be beneficial as a complementary analysis for univariate analyses to evaluate the adaptation process at the whole-brain level.

The present study demonstrated neuroplastic changes in the whole-brain macroscopic activity patterns induced by brain-computer interfacing in the first 2 h. Although the primary focus of the present study was to detect the differential interaction of human brain and classifiers at the early period, the difference elicited by long-term use is not mentioned in the study. While previous BCI studies have demonstrated the long-term co-adaptation is one successful strategy for efficient training (Perdikis et al., 2018; Silversmith et al., 2021), however, the limited amount of training period in the study did not elicit the adaptation of neural activity patterns by the adaptive classifier use. Such differences in the adaptation process depending on time scale are warranted in the further investigation.

Although the flexibility of the human brain enabled adaptation to model-based classifiers and partly to the *de novo*, the adaptive classifier did not elicit adaptation of neural activity patterns, manifested by the absence of any changes in geometric assessment at least the early stage of BCI operation training. It would be because the adaptive BCI enhanced its performance by classifier-side adaptation, that maximized the separability of two brain states for the current data by classifier reconfiguration. In contrast to the previous studies demonstrating co-adaptation of brain and classifiers using the trial-by-trial classifier adaptation (Wolpaw and McFarland, 2004; Orsborn et al., 2014), the block-by-block calibration procedure waived the previously optimized parameters and reconstructed an entirely new model, the classifier-side adaptation could have been dominant. As the user did not receive neurofeedback based on the constant rule (i.e., parameters of the classifier) putatively because of the abrupt changes in the classifier parameters as well as the CSP features, it could have achieved the high separability of two brain states without engaging cortical adaptation process yet interfered the user’s attempt to adapt to the classifier. In summary, fixation of the classifier plane is an essential element for inducing neural plasticity via a brain-computer interaction based on macroscopic neural populational activities, and adaptation to a BCI based on unnaturalistic features without the instruction of suitable strategies for control is partly possible in the initial stage of BCI operation training. This demonstration may in part explain human adaptability to external environment that continuously changes over time, underlying the flexibility of our motor performance.

## Synthesis

Reviewing Editor: Mark Laubach, American University Decisions are customarily a result of the Reviewing Editor and the peer reviewers coming together and discussing their recommendations until a consensus is reached. When revisions are invited, a fact-based synthesis statement explaining their decision and outlining what is needed to prepare a revision will be listed below. The following reviewer(s) agreed to reveal their identity: Serafaim Perdikis.

Thanks for sending your study to eNeuro. The same two reviewers reviewed your revised manuscript. One was satisfied with the revisions. The other feels strongly that one final round of revisions is necessary. The other reviewer agreed with this assessment after reading the reviews. Please address all points raised below in your revised manuscript. Thank you.

Advances the Field: I think this study’s main contribution to the field is to consider how learning can drive changes in brain-wide neural activity (using the EEG signal), in contrast to previous using neural activity local to a particular brain region (e.g., using electrophysiology in M1).

Statistics: One of the key analyses/results in this paper regards Figure 7, where one of the questions is whether or not neural activity shows significant changes during “late” vs. “early” for the adaptive decoder. As described in my comments to the authors, it’s not clear how exactly this analysis was performed, but doing this the way it seems they might be doing it would not be consistent with the conclusion they make. Also more generally, for that same figure/analysis, it seems they are taking their inability to reject the null as positive evidence that the null hypothesis (neural activity does not adapt under the adaptive decoder) is true.

Comments to the Authors: I would first like to thank the authors for their substantial updates to the manuscript. I feel like all of my comments/questions on the initial submission were taken into account, and overall the manuscript is much clearer as a result. Specifically, the descriptions of the task, the authors’ hypotheses, and how those hypotheses are being tested by the BCI decoders used in the experiments were all much easier for me to follow in this version of the manuscript.

That being said, there are some major details regarding some of the key analyses (and the conclusions the authors’ make from those analyses) that now stand out to me. I think these would need to be addressed by the authors before the paper should be accepted. Briefly, these are 1) the validity of measuring tNorm and theta_p using the approximation (“V”) in the embedding space; 2) the need to consider subject-by-subject variability in performance when analyzing subject-by-subject changes in the neural metrics; 3) the exact ways in which tNorm and theta_p were computed for the adaptive decoder, given that its classifier changed every session; and 4) the authors’ conclusion that learning using the adaptive decoder is due to changes in the classifier, and not changes in the neural activity. These points are explained in more detail below.

Major comments (detailing points 1-4 above):

1. Both of the key neural metrics, tNorm and theta_p, involve the approximation “V” that is fit in the 3D tSNE space. It seems possible that V is not adequately representing how neural activity must change to improve behavior. For example, in Figure 7, many subjects show tNorm < 0 even in the “late” period. From my understanding, this either implies that i) most subjects are moving their neural activity in the opposite direction of the classifier, even after learning; or ii) that the approximation, “V”, of the classifier in the embedded space is not a very good approximation.

2. In figures 6-7, I wonder if it makes sense to only include subjects who did learn. this seems important because if one of the claims is “subjects learn the adaptive classifier, but not because the neural activity changed systematically,” then it seems important to only include those subjects who *did* improve performance when showing the results in figure 7. the reason I thought to ask this is as follows. in figure 4, where it’s clear that the across-subject average showed a lot of performance improvement using the adaptive classifier, but then in figure 6c, there is some across-subject variability in terms of how |tVec| changes; can this be explained by some of these subjects learning (e.g., those showing an increase in |tVec|, and a decrease in tNorm and theta_p), and others not learning? On a related note, if the authors take the above suggestion, it might make sense to assess average performance in the “early” vs. “late” periods, *for each subject*, to parallel the analyses made on the neural activity.

3. It would be helpful to understand more precisely how tNorm and theta_p are being computed for the adaptive classifier, in figure 7. Take tNorm, for example. My current understanding (which may be wrong) is that for each subject, tNorm is calculated separately for each session i, using the classifier from session i-1; then the tNorm is averaged across sessions 1-4 for the “early” period, and across sessions 13-16 for the “late” period. If this is true, this seems problematic for the authors’ intepretation that changes in performance for the adaptive classifier cannot be explained by *neural* adaptation. For this to be a valid conclusion, we would need tNorm to be calculated relative to the *same* baseline in both early and late (as it is for the other classifiers, where this is more easily done), in order to expect a decrease in tNorm. I recognize that this analysis is challenging for the adaptive classifier, but it is nevertheless important for the conclusion the authors make. (Perhaps one alternative would be to find V_i for the adaptive classifier across each session i, and if the variance across V_i’s is low enough, perhaps you could approximate these V_i’s as the average, V, and use that V to compute tNorm? I’m not sure if this would work though.)

4. The authors conclude that “Further, the performance improvement elicited by the adaptive classifier was mainly driven by the classifier-side adaptation rather than the cortical adaptation as suggested by the absence changes in any of brain-side metrics.” Overall, I’m not sure there’s much evidence of this conclusion beyond the absence of a significant result in Figure 7c. Even so, I don’t think one can intepret the absence of evidence to reject a null hypothesis as positive evidence _for_ the null hypothesis.

Minor comments:

- the authors refer to “brain-side” and “machine-side” adaptation, which are not terms I’m familiar with, and took me a while to figure out. In the abstract and intro, perhaps the authors could rephrase these ideas using plain language

- very early in the methods, it would be helpful if the authors could describe the subject’s task in plain language; e.g., something along the lines of “the subjects were instructed to ___.” I say this because there are many references to different effectors (range grasp aperture; angle of tail; “rest” vs. “imagine”) but I have no idea, at this point in the methods, what these are referring to. reading further, I think my confusion would be greatly helped by moving the “Experimental procedure” section before the “Online processing...” section (and rewriting where necessary if some terms have not been introduced yet)

- the methods are much improved, in terms of explaining the performance definition, and the classifiers used. however, it’s still unclear to me exactly how these components (ERD, the classifier, and neural activity) are related. specifically, in the equation for ERD–is it that the power, P(t), is somehow a function of the recorded EEG activity and the parameters of the classifier? If so this should be explained, to help readers who are unfamilar with EEG BCI tasks (like myself!)

- related to the above, it would be helpful to present some quantity, z(t), which we can think of as the neural activity at time t. From what is later presented in the methods, I believe z(t) has dimensionality of 645? Then the relation between P(t) and z(t) can be described explicitly, which would make the link between ERD (performance) and neural activity more clear

- the description of the tasks, and what the subject is trying to do in each case, is much clearer, thank you!

- in each block with the same classifier, were the classifiers different? e.g., if blocks 1 and 6 both used the model-based classifier, could the subject use the same strategy to succeed at either?

- in the “Evaluation of BCI performance,” it would be helpful to state up front why you wanted to perform the analysis you’re about to describe—e.g. “To summarize whether each participant’s performance improved over a session for each classifier, we ...” Similar note for the section “Feature extraction of EEG-dataset...”

- the descriptions of tNorm and tVec are much clearer and better motivated—thank you!

- the ‘d’ in the equation for the classifier should be ‘b’ (∼line 354)

- “ordinal least squares” -> “ordinary least squares”

- “For adaptive classifiers, as the classifier plane was obtained from the 2nd session, the comparison was conducted from the 2nd session across all statistical tests” (line 396): can the authors explain what this means, “from the 2nd session”? do you mean that only the 2nd session defines the “early” period for the adaptive classifier?

- can the authors remind the reader in the results how the “early” and “late” period are defined? (line 447)
